# CD8^+^CXCR5^+^ T Cells Regulate Pathology in the Genital Tract

**DOI:** 10.1155/2013/813238

**Published:** 2013-01-10

**Authors:** Janina Jiang, Cheryl I. Champion, Bo Wei, Guangchao Liu, Kathleen A. Kelly

**Affiliations:** ^1^Department of Pathology and Laboratory Medicine, David Geffen School of Medicine, 10833 Le Conte Avenue, Los Angeles, CA 90095, USA; ^2^National Vaccine and Serum Institute, China National Biotec Group (CNBG), Beijing 100029, China; ^3^California NanoSystems Institute, University of California, 570 Westwood Plaza, Los Angles, CA 90095, USA

## Abstract

We have identified a CD8^+^CXCR5^+^ T cell that prevents the development of oviduct dilation following *C. muridarum* genital infection. Phenotypic studies show that CD8^+^CXCR5^+^ cells express markers of T regulatory cells (FoxP3, CD25, and GITR) but do not express a necessary component of cytotoxic cells (perforin). *Cxcr*5^−/−^ mice have significantly lower numbers of CD8^+^ cells and lack the CD8^+^CXCR5^+^ population while the total number of CD4^+^ cells is equivalent between mouse strains. The transfer of CD8^+^ splenocytes from WT mice reduces the oviduct dilation seen in *Cxcr*5^−/−^ mice following *C. muridarum* infection. Future studies will investigate the mechanism by which this cell type regulates genital tract pathology.

## 1. Introduction

Pelvic inflammatory disease (PID) is defined as inflammation of the uterus and/or fallopian tubes and is induced by a number of organisms following sexual transmission. *Chlamydia trachomatis* is the most common reportable sexually transmitted infection (STI) and is responsible for >1 million cases in the US and approximately 92 million cases worldwide each year [1, 2]. Genital infection can lead to immune-mediated damage of the female reproductive organs and serious reproductive disability, including PID that can result in chronic pelvic pain, ectopic pregnancy, and infertility [3]. The risk of developing infertility increases by 40%–70% following reinfection [4]. The reinfection rate is approximately 13% and occurs within 6 months [5]. Delivery of treatments designed to reduce the local inflammation and prevent fibrotic disease to infected individuals may be a viable approach for further reducing PID and the costs associated with its treatment.

Regulatory T cells (Tregs) are comprised of multiple subsets of T cells that suppress other T cells from engaging in detrimental immune responses [6]. Tregs are broadly categorized as natural or inducible. Natural Tregs induce tolerance, delete autoreactive T cells, and dampen inflammation during an autoimmune reaction [7–13]. Inducible Tregs arise during infections in response to the infectious process to restore the homeostatic environment. In some cases, Tregs can be actively induced by the pathogen and promote pathogen survival by preventing elimination [14]. Tregs have also been shown to protect mucosal surfaces of the intestine from inflammation [15]. The linage can be phenotypically identified by the Foxp3 transcription factor [16]. The most widely studied subset is phenotypically defined as CD4^+^CD25^+^FoxP3^+^. This subset has been shown to indirectly prolong microbial growth by interfering with the priming of naive or unstimulated T cells [17].

CD8 cells also have suppressive activity and have been identified with and without FoxP expression to include the following: CD8^+^CD25^+^FoxP3^+^, CD8^+^CD45RC^low^FoxP3^+^, CD8^+^CD28^−^FoxP3^−^, CD8^+^CD122^+^FoxP3^−^, and CD8*αα* ^+^FoxP3^−^ as in [18, 19]. Recently CD8^+^ Tregs have emerged as preventing the pathogenic cascade leading to the onset of fibrotic disease in primary biliary cirrhosis and systemic sclerosis [20]. In this study, we identified a CD8^+^ cell that coexpresses CXCR5 and regulates genital tract pathology *in vivo *following infection with *C. muridarum*. 

## 2. Materials and Methods

### 2.1. Animals, Chlamydia, and Infection of Mice

A breeding colony was established with *Cxcr*5^−/−^ mice (8 generations in C57BL/6) obtained from Martin Lipp, Delbrück-Center for Molecular Medicine, Berlin, Germany. Female C57BL/6 mice, 5-6 weeks old (Harlan Sprague-Dawley, Indianapolis, IN, USA) were housed according to American Association of Accreditation of Laboratory Animal Care guidelines. Animal experimental procedures were approved by the UCLA Institutional Animal Care and Use Committee. *Chlamydia muridarum* was grown on confluent McCoy cell monolayers, purified on Renografin gradients and stored at −80°C in sucrose-phosphate-glutamine buffer (SPG) as previously described [21]. Mice were hormonally synchronized by subcutaneous injection with 2.5 mg of medroxyprogesterone acetate (Depo Provera, Upjohn, Kalamazoo, MI, USA) in 100 *μ*L saline 7 days prior to a vaginal challenge with 1.5 × 10^5^ infection forming units (IFUs) of *C. muridarum* under anesthetization. Depo Provera drives mice into a state of anestrous and eliminates the variability in the rate and severity of infection due to the estrus cycle. Infection was monitored by measuring IFUs from cervical-vaginal swabs (Dacroswab Type 1, Spectrum Laboratories, Rancho Dominguez, CA, USA) as described [21]. 

### 2.2. Histology

The genital tracts (GTs) were removed and, fixed in 10% formalin overnight, followed by 70% ethanol. Tissues were embedded en bloc in paraffin, sectioned (5 mm), and stained with hematoxylin and eosin. Tissue blocks were cut transversally from the ovary, and sections were collected at the beginning of the transitional region between ovary and oviduct. A veterinarian pathologist scored 2 sections from the right and left oviducts of each mouse for luminal dilation; 0 = luminal oviduct size of naïve mice, 1+ = mildly increased luminal oviduct size, 2+ = moderately increased luminal oviduct size, 3+ = severely increased luminal oviduct size, and 4+ = severely increased luminal oviduct size in greater than 75% of oviducts.

### 2.3. Lympholyte Isolation and FACS Identification

Spleen (Spl) and mesenteric lymph nodes (MLN) were harvested from individual mice. Single cell suspensions were attained by dissociating cells within the organs. Lymphocytes were incubated in RPMI 1640 in the presence of PMA and ionomycin. Brefeldin A (Sigma-Aldrich, St. Louis, MO, USA) was added 4 hr before the end of the culture period. The cells were then stained with fluorochrome-labeled antibodies against CD3 (clone 145-2C11), CD4 (clone GK1.5), CD8*β* (clone eBioH35-17.2), CXCR5 (clone 2G8), CD25 (clone PC61.5), GITR (DTA-1), CD122 (clone TM-beta 1), CD127 (clone A7R34), TCR*β* (clone H57-597), TCR*γδ* (clone eBioGL3), *α*-GalCer-CD1d-tetramer a gift from Mitchell Kronenberg [22], and irrelevant control abs (eBioscience, San Diego, CA or BD Biosciences, San Jose, CA, USA) for 20 min on ice. After being washed, the cells were incubated with Cytofix/Cytoperm (eBioscience) for 1 hr and stained with fluorochrome-conjugated FoxP3 (clone FJK-16s) for 20 min on ice, washed again, and resuspended in Cell Fix solution (eBioscience). Flow cytometry was performed on a fluorescence activated cell sorting analyzer equipped with a 488 nm argon laser and CellQuest software (FACScan; BD Biosciences). The instrument was calibrated with beads (CaliBRITE; Becton Dickinson), using AutoCOMP software. Dead cells were excluded on the basis of forward angle and 90° light scatter, and 10,000 gated cells were analyzed for each sample.

### 2.4. Cell Sorting and Adoptive Transfer

Lymphocytes were isolated from the spleens of infected and uninfected mice as described above. Cells were stained for CD3 and CD8 and sorted for the CD3^+^CD8^+^ population using FACSAria cell sorter (BD Bioscience) in the UCLA FACS Core Labaratory. The purity of the sorted population was 99%. Cells were resuspended in saline and 1 × 10^6^ cells were intravenously injected into the recipient through the tail vein. At the time of transfer, the mice were infected with *C. muridarum* as described above. These mice were also synchronized with medroxyprogesterone acetate 7 days prior to infection as described above.

### 2.5. Statistics

The percentage of CD4 and CD8 cells, oviduct luminal dilation scores, and IFU counts from WT and *Cxcr*5^−/−^ mice were compared using Student's *t*-test, Kruskal-Wallis, and ANOVA tests, respectfully, with software from GraphPad Software, Inc. (La Jolla, CA, USA). Groups were considered statistically different at *P* values of <0.05.

## 3. Results and Discussion

### 3.1. Phenotypic Characterization of CD8^+^CXCR5^+^ Cells

**Figure 1:**
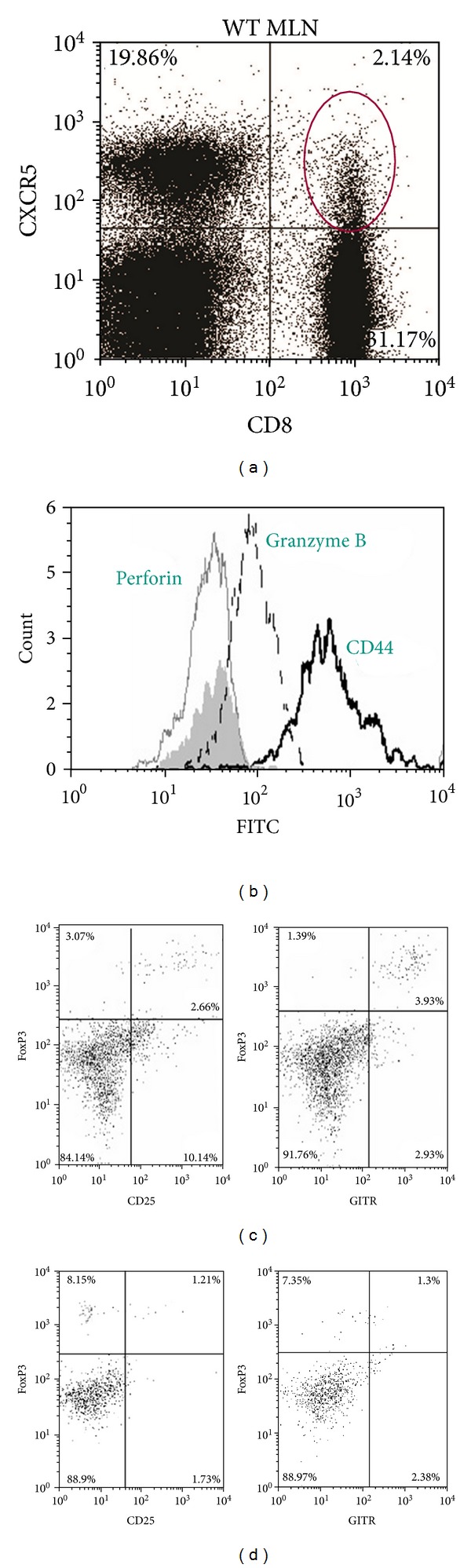
CD8^+^CXCR5^+^ cells express the phenotype of Tregs. MLNs were isolated from C57BL/6 WT mice 7 days after genital infection with *C. muridarum* and stained with CD3, CD8, CXCR5, and various markers of cytotoxic and regulatory T cells. (a) The dotplot shows the percentage of CD8 and CXCR5 on CD3^+^ cells. (b) Histogram of CD3^+^CD8^+^CXCR5^+^ cells showing the individual expression of perforin (gray line), granzyme B (dash line), and CD44 (black line). Irrelevant control antibody stained cells appear as a shaded histogram. (c) Dotplot showing the percent of CD3^+^CD8^+^CXCR5^+^ cells expressing of FoxP3 and CD25 or FoxP3 and GITR following stimulation of spleen cells from mice during genital infection with *C. muridarum*. (d) Dotplot showing the percent of CD3^+^CD8^+^CXCR5^+^ cells expressing of FoxP3 and CD25 or FoxP3 and GITR following stimulation of spleen cells from naive mice.

We identified a population of CD8^+^ cells that expressed CXCR5 and comprised 2%-3% of CD8 T cells in the MLN (Figure 1(a)) and spleen (data not shown). The percentage of CD8^+^CXCR5^+^ cells in the MLN and spleen did not change in a naive mice or following *C. muridarum* genital infection (data not shown). Phenotypic analysis found that these cells were memory cells by high expression of CD44. Analysis of cytotoxic markers, granzyme B and perforin, showed that these cells did not express perforin. Although granzyme B was expressed, these cells are not considered cytotoxic since perforin is required for entry of granzymes into the cell cytoplasm as shown in mice deficient in perforin [23–25]. In addition, cytotoxic memory cells express high levels of both granzyme B and perforin (Figure 1(b)) [26]. Examination of markers of Tregs showed a subpopulation of CD8^+^CXCR5^+^ cells that expressed CD25, Foxp3 and GITR (glucocorticoid-induced tumor necrosis factor receptor family related gene) upon stimulation from mice during a genital infection with *C. muridarum* (Figure 1(c)). Analysis of CD122 or CD127 was negative (data not shown). Interestingly, induction of this population could not be found on stimulated cells from naive mice (Figure 1(d)). This finding indicates that a subpopulation of CD8^+^CXCR5^+^ cells express phenotypic markers associated with Tregs and not cytotoxic cells. 

### 3.2. CD8^+^CXCR5^+^ Population Is Absent in *Cxcr*5^−/−^ Mice

**Figure 2:**
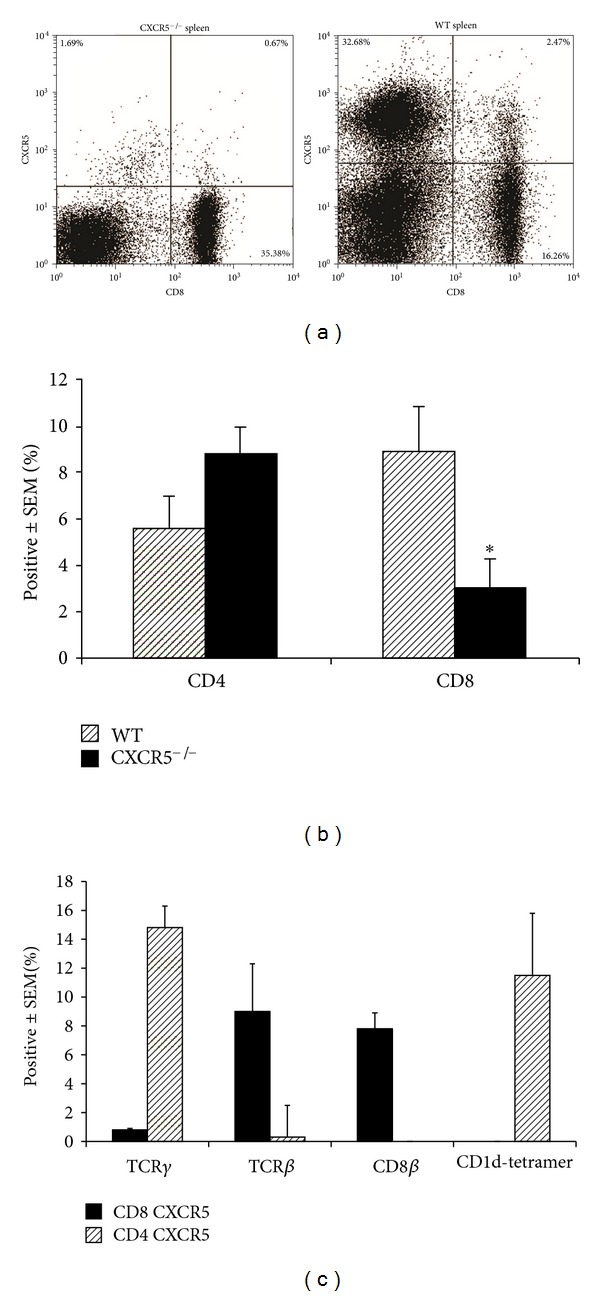
*Cx* *cr*5^−/−^ mice lack CD8^+^CXCR5^+^ cells. Spleen cells were isolated from C57BL/6 WT mice 7 days after genital infection with *C. muridarum* and stained with CD3, CD4 or CD8, CXCR5, and markers of T cells; TCR*γδ*, TCR*β*, CD8*β* or NKT cells; CD1d-tetramer. (a) The dotplot shows the percentage of CD8 and CXCR5 of CD3^+^ cells from *Cxcr*5^−/−^ and WT mice. (b) The percent of CD4 or CD8 populations from groups of 6 mice were compared between *Cxcr*5^−/−^ and WT mice by Student's *t*-test. The ∗ indicates *P* < 0.01. (c) The percentage of CD4 or CD8 cells expressing markers of T cells; TCR*γ*, TCR*β*, CD8*β* or NKT cells; CD1d-tetramer.

To further characterize the phenotype and examine the function of CD8^+^CXCR5^+^ cells, we evaluated the CD8 population in *Cxcr*5^−/−^ mice. As expected, we did not find any CD8^+^CXCR5^+^ cells in mice lacking CXCR5 (Figure 2(a)). *Cxcr*5^−/−^ mice had slightly less CD3^+^ T cells (data not shown) but had similar numbers of CD4 cells. In addition, the mice had significantly less CD8 cells compared to WT mice (Figure 2(b)). Further analysis showed that these cells were CD8 T cells by expression of the TCR and CD8 *β* chain and not a *γδ* T cell or NKT cell that could express CD8 (Figure 2(c)). 

### 3.3. Adoptive Transfer of CD8^+^ Cells Reversed Oviduct Dilation after *C. muridarum* Infection in *Cxcr*5^−/−^ Mice

**Figure 3:**
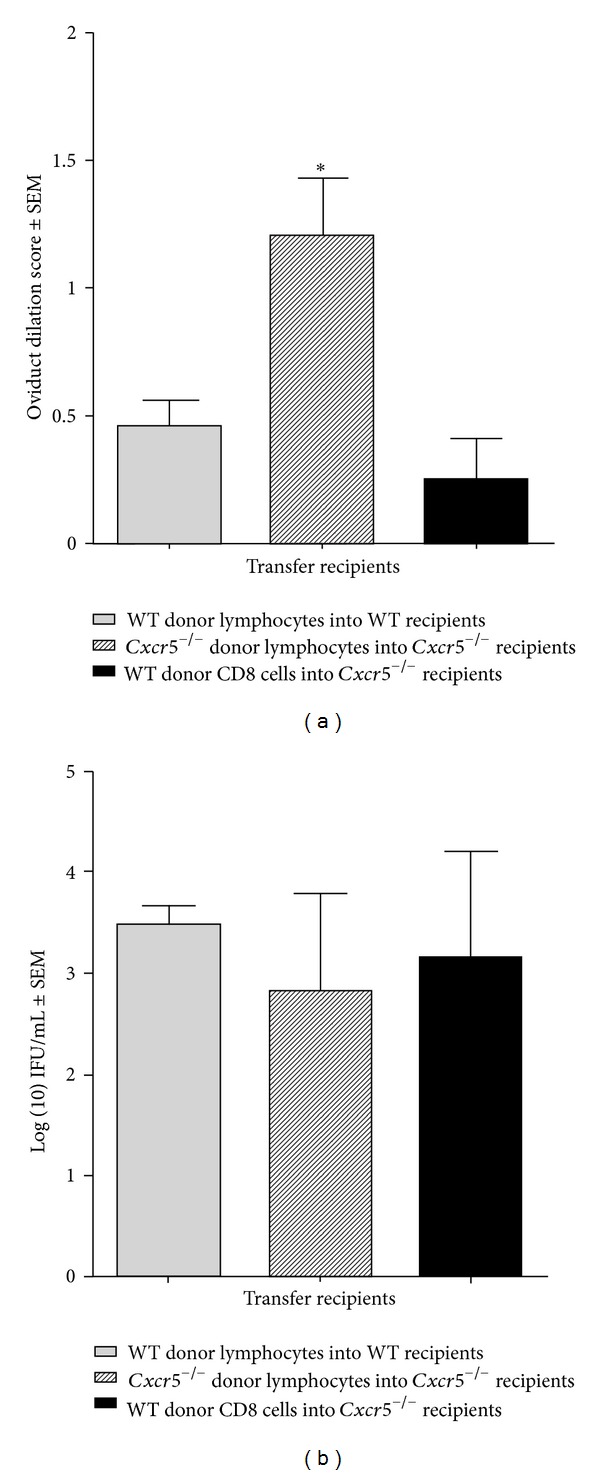
Adoptive transfer of CD8^+^ cells reversed oviduct dilation late after *C. muridarum* infection in *Cxcr*5^−/−^ mice. (a) Donor *Cxcr*5^−/−^ and WT mice were infected with *C. muridarum* and the spleens were harvested 7 days later and lymphocytes were obtained by dissociating cells within the organs. Donor C57BL/6 WT mice were infected with *C. muridarum* and the spleens harvested 7 days after infection. CD8^+^ cells were purified by FACS sorting for expression of CD8. *Cxcr*5^−/−^ lymphocytes and WT-CD8^+^ were adoptively transferred (1 × 10^6^) into *Cxcr*5^−/−^ mice as indicated. Genital tracts were harvested 7 weeks later and hemetoxylin and eosin staining was performed and scored. Oviducts were scored individually and were compared between recipient groups using Kruskal-Wallis test. Transfer groups are, WT donor into WT recipient; *n* = 6 mice (12 oviducts), *Cxcr*5^−/−^ donor into *Cxcr*5^−/−^ recipient, *n* = 6 mice (12 oviducts), and WT-CD8 donor into *Cxcr*5^−/−^ recipient, *n* = 2 mice (4 oviducts). The bars indicate the mean ± SEM and *P = 0.02. (b) Recipient mice were vaginally swabbed 9 days after infection and all groups were compared by one-way ANOVA. There were no differences between groups. The bars indicate the mean IFU ± SEM.

WT and *Cxcr*5^−/−^ mice were intravaginally infected with *C. muridarum* and splenic lymphocytes were purified 7 days after infection. The lymphocytes or purified CD8^+^ cells were adoptively transferred into WT or *Cxcr*5^−/−^ recipients. The genital tracts were harvested seven weeks after infection, hematoxylin and eosin stained, and scored by a veterinarian pathologist. We found that the adoptive transfer of WT lymphocytes into WT recipients had significantly reduced oviduct dilation compared to *Cxcr*5^−/−^ mice given *Cxcr*5^−/−^ lymphocytes. Further, *Cxcr*5^−/−^ mice given purified CD8^+^ cells from WT mice also showed significantly reduced oviduct dilation (Figure 3(a)). It is possible that the transfer of CD8 effector cells could account for a reduction in oviduct dilation by perhaps reducing bacterial burden. However, there was no difference in bacterial burden in the GT on days 9 following infection between *Cxcr*5^−/−^ mice given WT-CD8 cells or *Cxcr*5^−/−^ lymphocytes or WT mice given WT lymphocytes (Figure 3(b)). Taken together, these data strongly suggest that CD8^+^CXCR5^+/+^ (WT mice) prevent or interfere with the development of oviduct dilation following *C. muridarum* genital infection.

## 4. Conclusions

Our study describes the identification of CD8^+^CXCR5^+^ cells that possess the ability to regulate oviduct dilation that occurs following the immune response to *C. muridarum* genital infection. This study highlights the finding that the CD8^+^ population is comprised of multiple subsets with differing function. Murthy et al., showed that CD8 cells secreting TNF*α* cause oviduct dilation and hydrosalpinx [27]. Individual differences in PID or infertility may be influenced by the proportions of CD8^+^ cell subsets. The mechanism whereby regulation of genital tract pathology occurs is not known. However, a small percentage of CD8^+^CXCR5^+^ cells express markers of Tregs; FoxP3, CD25, and GITR but not perforin, a marker of cytotoxic cells, and suggest that these cells function as T regulatory cells.

Our data show that adoptive transfer of CD8^+^CXCR5^+^ cells prevent oviduct dilation following genital infection with *C. muridarum *and suggests that this cell type is necessary for preventing oviduct dilation following genital infection. However, CD8^+^ cells with suppressive activity require IL-10^+^ APC for expansion [28]. IL-10^+^ APC have been shown to reduce the number of antichlamydial Th1 cells that develop and this results in a prolonged infection [29]. Others have suggested that there are a number of immune responses operating in different phases of the immune response and this could possibly explain the conflicting role of IL-10^+^ APC during *C. muridarum* infection.

A population of CD8 cells that also expresses CXCR5 has been reported but their function has not been identified [30]. There are a number of subsets of CD8^+^ Tregs that have been identified and include the following: CD8^+^CD25^+^FoxP3^+^, CD8^+^CD45RC^low^FoxP3^+^, CD8^+^CD28^−^FoxP3^−^, CD8^+^CD122^+^FoxP3^−^, and CD8*αα*FoxP3^−^ [18]. We found that a small percentage of CD8^+^CXCR5^+^ cells expressed FoxP3 upon antigen stimulation but did not express CD122 or CD8*αα*. The mechanism of suppression of the CD8*αα* subset has been reported. This subset is restricted to nonclassical MHC class Ib molecules, Qa-1, and has been found to suppress autoantibody formation and development of systemic lupus erythematosus (SLE-) like disease through inhibition of T_FH_ [31]. Although CXCR5 expression does not prevent entry into germinal centers, the *Cxcr*5^−/−^ mice used in our study do not have T_FH_ cells or germinal centers and have reduced levels of immunoglobulins [32, 33]. Likewise we also find a reduction in antichlamydial IgM and IgG levels (data not shown). Additionally, regulation by the CD8*αα* subset depended on perforin and perforin was not found on activated CD8^+^CXCR5^+^ cells in this study confirming that CD8^+^CXCR5^+^ identified in this study are not the CD8*αα* expressing cell described previously. 

T regulatory cells primarily effect T cells and/or dendritic cells and have four mechanisms by which they suppress immune responses. T regulatory cells secrete inhibitory cytokines such as IL-10 and TGF*β*, induce apoptosis of target cells using the granzyme-perforin pathway, deprive effector T cells of IL-2 which leads to apoptosis of effector T cells, and inhibit dendritic cell function [19]. Our study has found that the granzyme-perforin pathway is unlikely to be used by CD8^+^CXCR5^+^ based on a lack of expression of the effector molecule, perforin. It would appear unlikely that suppressive cytokines are secreted since the resolution of infection is similar between *Cxcr*5^−/−^ and WT mice and IL-10 secretion has been shown to prolong the course of infection [29]. Future studies will determine whether CD8^+^CXCR5^+^ function as T regulatory cells and act directly on T cells or on another cell type such as dendritic cells.
